# Circulating Cell-Free Mitochondrial DNA as a Novel Biomarker for Intra-Amniotic Infection in Obstetrics: A Pilot Trial

**DOI:** 10.3390/jcm13164616

**Published:** 2024-08-07

**Authors:** Sebastian Zeiner, Peter Wohlrab, Ingo Rosicky, Regina Patricia Schukro, Klaus Ulrich Klein, Johann Wojta, Walter Speidl, Herbert Kiss, Dana Anaïs Muin

**Affiliations:** 1 Department of Anaesthesia, Intensive Care Medicine and Pain Medicine, Medical University of Vienna, 1090 Vienna, Austria; 2 Department of Obstetrics and Gynaecology, Division of Obstetrics and Feto-Maternal Medicine, Medical University of Vienna, 1090 Vienna, Austriadana.muin@meduniwien.ac.at (D.A.M.); 3 Department of Internal Medicine 2, Medical University of Vienna, 1090 Vienna, Austria

**Keywords:** biomarker, intra-amniotic infection, premature preterm rupture of the membranes (PPROM), mtDNA

## Abstract

**Background/Objectives**: Intra-amniotic infection (IAI) is a rare but serious condition with potential complications such as preterm labor and intrauterine fetal death. Diagnosing IAI is challenging due to varied clinical signs. Oxidative stress and mitochondrial dysfunction have been hypothesized to evolve around IAI. This study focused on measuring circulating mtDNA levels, a proposed biomarker for mitochondrial dysfunction, in maternal serum and placenta of women with confirmed IAI and healthy controls. **Methods**: 12 women with confirmed IAI (IAI group) were enrolled following premature preterm rupture of the membranes (PPROM) and compared to 21 healthy women (control group). Maternal blood was obtained two weeks pre-partum and peripartum; furthermore, postpartum placental blood was taken. In the IAI group, maternal blood was taken once weekly until delivery as well as peripartum, as was placental blood. Circulating cell-free mtDNA was quantified by real-time quantitative PCR. **Results**: Upon admission, in the IAI group, mean plasma mtDNA levels were 735.8 fg/μL compared to 134.0 fg/μL in the control group (*p* < 0.05). After delivery, in the IAI group, mean mtDNA levels in the placenta were 3010 fg/μL versus 652.4 fg/μL (*p* < 0.05). **Conclusions**: Circulating cell-free mtDNA could serve as a valuable biomarker for IAI prediction and diagnosis. Future research should establish reference values for sensitivity in predicting IAI.

## 1. Background

Mitochondrial DNA (mtDNA) plays a crucial role in cellular energy production and is increasingly recognized as a biomarker in various diseases. Mitochondrial dysfunction underlies many diseases of oxidative stress, and mitochondrial DNA (mtDNA) content has been discussed as a biomarker [1,2].

According to the endosymbiotic hypothesis, mtDNA is evolutionarily derived from the circular genome of bacteria [3]. Its structure consists of a circular double-stranded DNA containing CpG DNA repeats, and it also codes for N-formyl peptides [4]. Due to its similarities to bacterial DNA, there is growing evidence that mitochondrial DNA is a potent damage-associated molecular pattern (DAMP) with the ability to trigger the inflammation cascade. This activation of inflammation was recently reported by Krychtyuk and coworkers, showing that mitochondrial DAMPs, similar to microbial pathogen-associated molecular patterns (PAMPs), directly bind toll-like receptors (TLR) [5]. Unmethylated CpG from mitochondrial DNA acts as a ligand for TLR-9, leading to the activation of NF-κB (nuclear cactor kappa B)and MAPK (mitogen-activated protein kinase) pathways [6]. Normal mtDNA levels during pregnancy are mostly close to non-pregnant levels. The two published studies concerning mtDNA during pregnancy have conflicting results. One has shown a progressive decrease in maternal blood mtDNA content through the first, second, and third trimesters compared to that in nonpregnant women [7]. The other has shown no difference during early pregnancy compared to non-pregnant women and a rise of mtDNA in late pregnancy [8].

Circulating mitochondrial DNA levels are inversely related to placenta oxygen partial pressure in the umbilical vein and directly related to and therefore a potential early biomarker for intra-uterine growth restriction (IUGR) as well as in pre-eclampsia [7,9]. Similarly, increased mtDNA levels are observed in women with pre-pregnancy obesity and GDM, indicating chronic inflammation and a higher risk of complications [10]. This indicates that increased mtDNA levels could serve as an early biomarker for several conditions as well as intra-amniotic infection (IAI).

Intra-amniotic infection (IAI) is an inflammation and/or infection of any combination of the amniotic fluid, placenta, fetus, fetal membranes, or decidua. IAI commonly results from an infection that ascends from the genital tract [11,12] and accounts for 50% of deliveries before 30 weeks of gestation. IAI may be present in up to 95% of women delivering between 21 and 24 weeks of gestation [13,14,15]. The presence of infectious agents in the chorioamnion triggers a maternal and fetal inflammatory response characterized by the release of a combination of proinflammatory and inhibitory cytokines and chemokines into the maternal and fetal compartments. The inflammatory response may produce clinical chorioamnionitis and/or lead to prostaglandin release, ripening of the cervix, membrane injury, and labor at term or premature birth at earlier gestational ages. In addition to preterm delivery, IAI is associated with several maternal and neonatal morbidities such as postpartum uterine atony, neonatal sepsis, pneumonia, or meningitis, and maternal infection (sepsis, peritonitis, endometritis), as well as neonatal seizures, cerebral palsy, respiratory distress, and even fetal and neonatal death [11,16,17,18,19]. The presence of infectious agents triggers an inflammatory response that may affect mtDNA levels.

The diagnosis of IAI is confirmed following amniocenteses, or postpartum, by the histology of the placenta, cord, or membranes. Often IAI is suspected upon clinical symptoms, such as maternal fever, purulent vaginal discharge, or fetal tachycardia [11,12,20]. As the diagnosis of IAI is a challenge, and its effect on neonatal and maternal health is detrimental, several studies have tried to identify biomarkers for early diagnosis of IAI, none of which have yet led to clinical application [21].

In conclusion, increased cell-free mitochondrial DNA could represent a pathogenic trigger for inflammation independent of pathogens and simultaneously be a valuable early biomarker for IAI that can help guide therapeutic interventions such as antibiotic treatment [22]. The objective of this study was to investigate maternal serum mtDNA levels in women with IAI compared to those in healthy pregnant women with the aim of identifying a possible biomarker for precise future diagnosis of IAI.

## 2. Materials and Methods

This study was conducted as a prospective controlled observational trial at the Department of Obstetrics and Feto-maternal Medicine at the Medical University of Vienna between January 2018 and July 2019. We included women admitted with preterm premature rupture of membranes (PPROM) between 22 and 28 + 0 weeks of gestation who met the diagnostic criteria for intra-amniotic infection (IAI) as the intervention group. The control group comprised women who were scheduled for elective cesarean section and were recruited during routine check-ups approximately two weeks prior to their planned delivery. All participants were aged between 18 and 45 years and provided signed and dated informed consent. 

According to the ACOG Guidelines, suspected intra-amniotic infection was based on clinical criteria (i.e., maternal intrapartum fever and/or maternal leukocytosis, purulent cervical drainage, or fetal tachycardia. In all cases, we obtained a vaginal swab for gram stain and culture results. In rare cases, we conducted an amniocentesis to confirm IAI. Postpartum, placental histopathology was performed in all cases to confirm chorioamnionitis and/or funisitis [23].

### 2.1. Sample Size Estimation

This study was designed as a pilot investigation, and formal sample size calculations were not possible due to limited data in this area. However, based on findings from Colleoni et al. (2010), which demonstrated significantly higher mitochondrial DNA (mtDNA) levels in intrauterine growth restriction (IUGR) pregnancies compared to those in normal pregnancies (430 ± 158 vs. 144 ± 54, respectively) we estimated that a sample size of 10 patients per group would suffice to detect potential alterations in circulating cell-free mtDNA levels associated with preterm premature rupture of membranes (PPROM) and intra-amniotic infection (IAI) [7]. Given the anticipated incidence rates of PPROM and IAI, we aimed to enroll 15 patients in each group for this study. This approach aligns with similar studies that investigated biomarkers in obstetric complications [9,24].

This study was approved on 21 March 2017 by the Ethics Committee of the Medical University of Vienna (Chairperson: Prof. Martin Brunner). Approval Code: EK-Nr: 1115/2017. The study complied with the principles outlined in the Declaration of Helsinki and the guidelines of Good Scientific Practice and Good Clinical Practices. Written informed consent was obtained from all participants.

### 2.2. Sample Analysis

In the control group, 14 mL of maternal blood was taken two weeks before planned delivery, during delivery, and from the placenta postpartum. In the intervention group, to observe the progression, blood was taken at study inclusion, and additional blood samples were taken once every week until delivery.

After obtaining written informed consent and recording baseline demographic data (including age, current weight, and gestational age), immediately following delivery, blood was drawn into a 9 mL ethylenediaminetetraacetic acid (EDTA) tube (BD Vacutainer^®^, Plymouth, UK) to obtain platelet-poor plasma for the evaluation of mtDNA.

Placental blood was also collected in the same manner. All blood samples were centrifuged within 2 h of collection at 4 °C with a relative centrifugal force of 3000× *g* for 30 min. The samples were then stored at −70 °C until analysis to prevent degradation and avoid interference from repeated freeze–thaw cycles.

DNA in the platelet-poor plasma fraction was extracted using a Qigen DNeasy Blood Mini Kit (Qigen, Venlo, The Netherlands) on an automated QIAcube platform (Qiagen). Plasma copy numbers were measured by quantitative real-time polymerase chain reaction for a mitochondrial gene cytochrome c oxidase 2 using a LightCycler TaqMan Master (Roche, Basel, Switzerland) according to manufacturer’s instructions. Cycling conditions were the following: 10 min at 95 °C, followed by 60 cycles of 95 °C for 15 s and 60 °C for 30 s. The primers for cytochrome c oxidase 2 subunit (fwd 5′-CAAACATCACTTTGGCTTCG-3′, reverse 5′AGTCAAACCACATCTACAAAATGC-3′) were designed using the Roche Universal Probe Library Assay Design Centre (https://lifescience.roche.com, accessed on 3 October 2019). To establish a standard curve for quantifying mtDNA content, a mitochondrial isolation kit for cultured cells (Pierce, Thermo Fisher Scientfic, Waltham, MA, USA) was used to isolate mitochondria from smooth muscle cells. Circulating plasma mtDNA levels were then calculated as a mean of technical triplicates of the cytochrome c oxidase 2 subunit gene.

### 2.3. Statistical Analysis

Statistical analysis was performed with Microsoft Excel (Microsoft Corporation, Redmond, WA, USA) and GraphPad Prism 9.5.0 (GraphPad Software, Boston, MA, USA). The data were expressed as the mean (standard deviation, ±SD) or numbers (frequencies). First, the data were tested for normal distribution using the Kolmogorov–Smirnov test. Differences between groups were tested using the Mann–Whitney U test if the data were not distributed normally, and the *t*-test or the Kruskal–Wallis test if they were normally distributed. Differences were considered significant if *p* < 0.05.

The primary endpoint of our study was the copy numbers of circulating cell-free mitochondrial DNA in maternal and placental venous blood.

The secondary endpoint was the analysis of confounding variables associated with peri- and postpartum complications, such as age, body mass index, and comorbidities.

## 3. Results

A total of 33 women were included in this study (IAI group *n* = 12, Cont. *n* = 21). Patient characteristics are provided in Table 1, including age, weight, pre-pregnancy body mass index (BMI), and neonatal birth weight. Mean maternal age at delivery was 32.08 (±5.47) years in the IAI group compared to 32.14 (±5.30) years in the control group. Mean gestational age at delivery and mean birth weight was 26.25 (±1.55) weeks and 911.3 (±231) grams in the IAI group and 38.05 (±0.50) weeks and 3243 (±491) grams in the control group. BMI and maternal weight did not differ significantly between the two groups with 27.44 (±5.81) kg/m^2^ and 74.92 (±14.85) kg in the IAI group and 29.77 (±4.55) kg/m^2^ and 79.72 (±11.81) kg in the control group. There were no significant differences between the groups, except for neonatal birth weight and gestation age.

**Figure 1:**
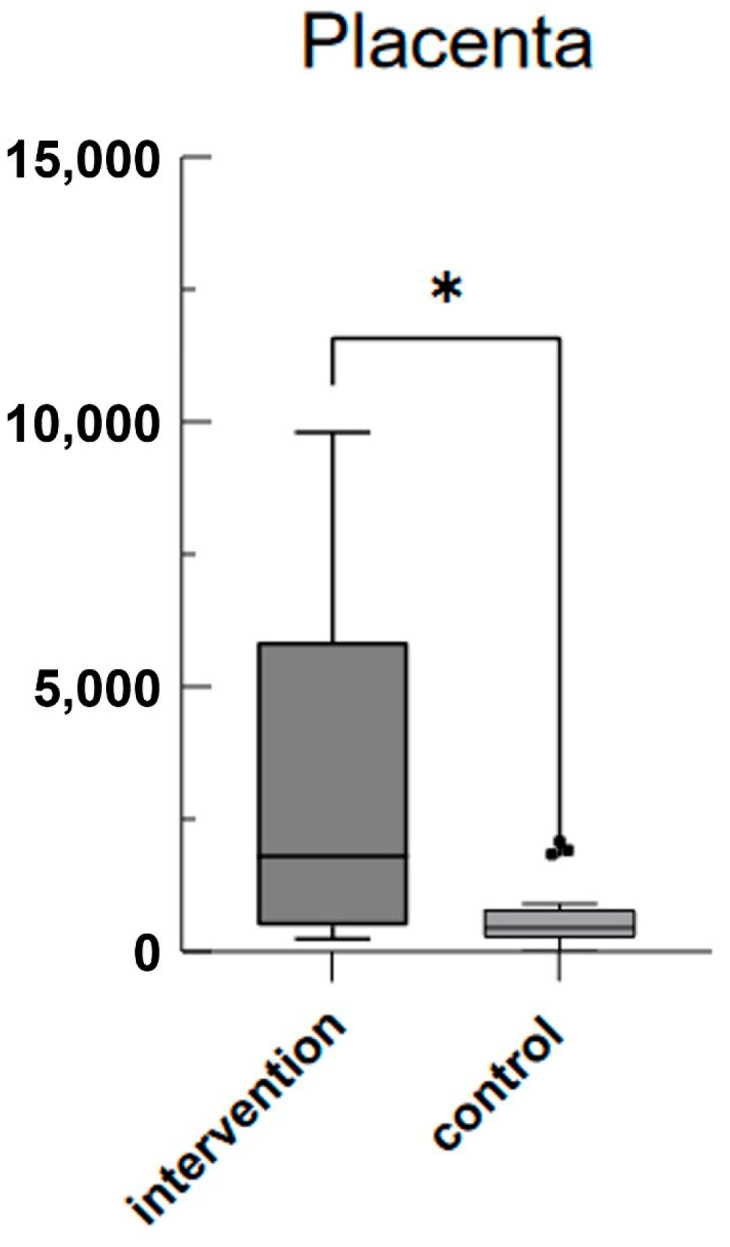
Mean mtDNA values in the IAI group (intervention) and control group in the placenta: box-and-whisker plot showing mean mtDNA values (fg/μL) in the placenta. The intervention group exhibited significantly higher median values compared to those of the control group (*).

**Figure 2:**
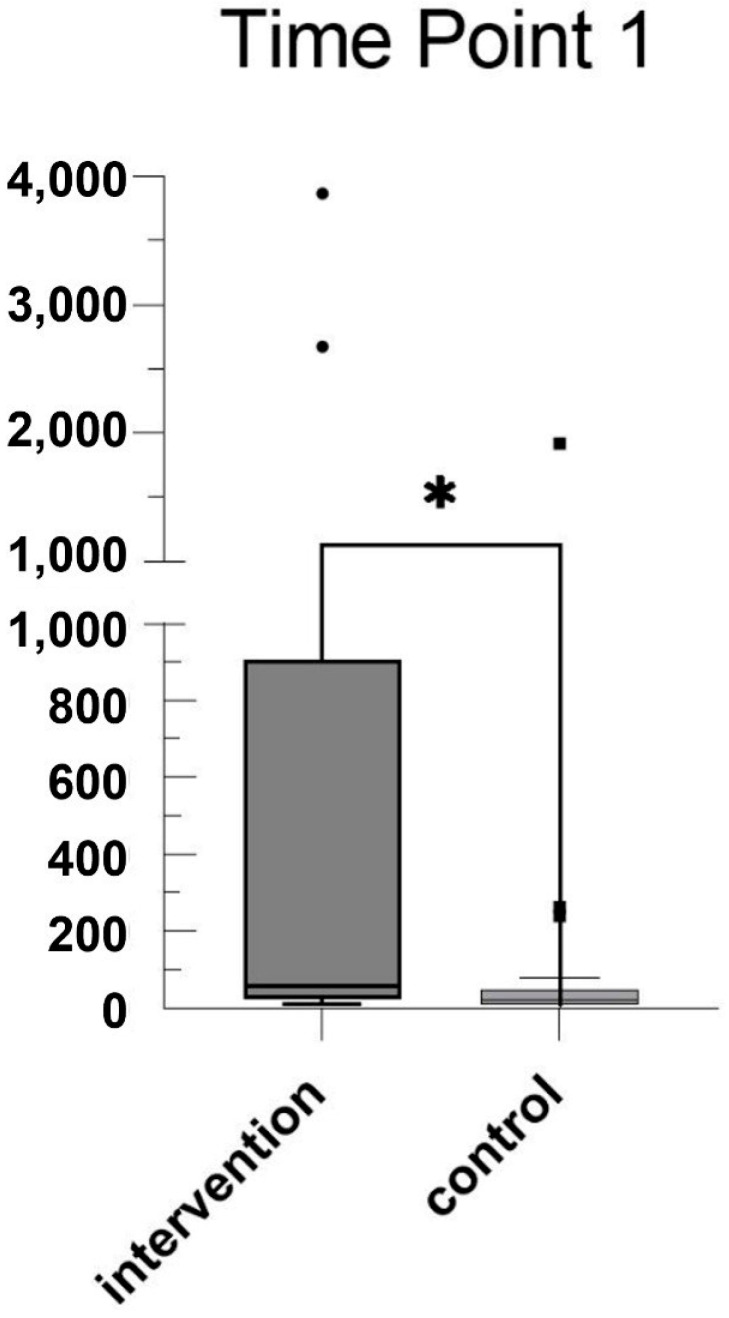
Mean mtDNA values in the IAI group (intervention) and control group before labor: box-and-whisker plot showing mean mtDNA values (fg/μL) before labor. The intervention group had higher median values and greater variability compared to those of the control group. Significant difference is indicated (*).

**Figure 3:**
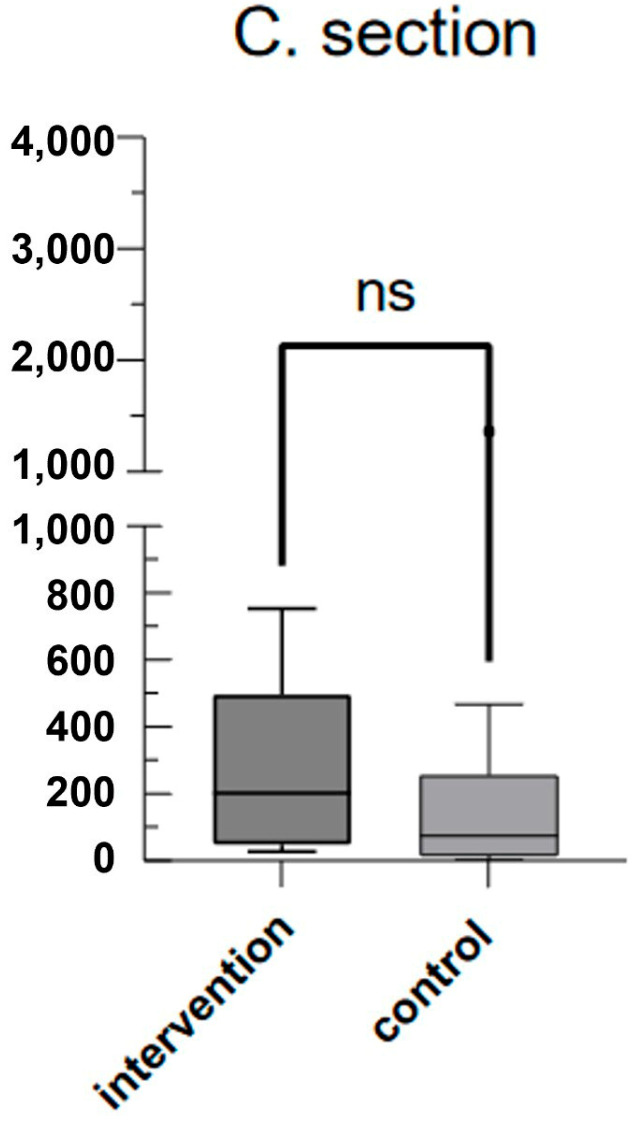
Mean mtDNA values in the IAI group (intervention) and control group during labor: box-and-whisker plot showing mean mtDNA values (fg/μL) during labor. The intervention group displayed slightly higher median values than the control group did, but the difference was not significant (ns).

As can be seen in Figure 1, mean plasma mtDNA levels at the time of IAI diagnosis (time point 1 (tp1)) were significantly higher with 735.8 fg/μL (SD ± 1373) compared to 134.0 fg/μL (SD ± 416.2) in the control group 2 weeks before scheduled caesarean delivery (*p* = 0.0348). Mean plasma mtDNA levels at the time of sectio were 290.1 fg/μL (SD ± 254.8) in the IAI group and 202.2 fg/μL (SD ± 322.6) in the control group (*p* = 0.095) (see Figure 2). Mean plasma mtDNA levels taken from the placenta after birth also differed significantly as seen in Figure 3, with 3010 fg/μL (SD ± 3502) in the IAI group and 652.4 fg/μL (SD ± 586.8) in the control group (*p* = 0.0241).

## 4. Discussion

In this study, we set out to detect mtDNA in women with and without confirmed IAI. The main finding of this study is that circulating cell-free mitochondrial DNA was significantly elevated in pregnant women with PPTOM associated with IAI compared to that in uneventful pregnancies. We demonstrated that mtDNA levels were increased before and after delivery. To our knowledge, this is the first prospective, controlled, and observational trial to measure maternal and placental mitochondrial DNA in the serum of women suffering from IAI.

As expected, due to the choice of a third-trimester control group, we observed a significantly shorter pregnancy duration and, consequently, a reduced birth weight in the IAI group. The high maternal mtDNA levels in intervention group are in accordance with recent studies showing increased circulating cell-free mtDNA in women suffering from pre-eclampsia or intra-uterine growth restriction. These studies suggest that a change in mtDNA concentrations in early pregnancy may be a predictor of the risk of developing pre-eclampsia. 

A recently published study by Cushen et al. showed that circulating cell-free mtDNA increases with advanced gestational age in healthy pregnant women [8]. These results created a possible opportunity to use concentrations of circulating mtDNA in normal pregnancy as a reference value for the development of clinical prognostic or diagnostic tests in pregnant women with, or at risk of developing, gestational complications. The data of Cushen et al. show that circulating cell-free mitochondrial DNA increases during late pregnancy in uneventful pregnancies. The observed rise in mtDNA in serum mirrors placental and fetal development in a physiological setting [8]. In our study, circulating cell-free mitochondrial DNA was significantly increased in women with IAI compared to that in uneventful pregnancies. A significant difference in IAI compared to physiologically increased cell-free mitochondrial DNA levels may lead to an optimistic view of mtDNA being a potential and sensitive biomarker. However, it is important to note that the findings of Cushen et al. stand in direct opposition to the findings of Colleoni et al., who found a reduction in maternal mtDNA in the third trimester compared to that in non-pregnant women and early pregnancy [7].

The recent change in the term of chorioamnionitis to Triple I (inflammation, infection, or both) was established to prevent physicians from including women with an isolated maternal fever into the diagnosis of chorioamnionitis. This points out that IAI is challenging to diagnose at an early stage due to its heterogenous clinical signs. A wrong diagnosis of IAI leads to increased exposure of infants to blood culture, lab tests, and the treatment with antimicrobial agents. Shatik et al. have shown that using a sepsis calculator would have reduced the number of infants needing lab tests and receiving antimicrobial agents to 12% without missing any of the positive-blood-culture early-onset sepsis infants. 

These results support the importance of finding new prognostic biomarkers to reduce the risk of harm to infants. Maternal blood has been the most extensively explored compartment to diagnose Triple I. Most biomarkers such as LDH, glucose, or CRP are too unspecific to predict Triple I. mtDNA has been described as a novel biomarker for fetal and placental development (studies). Circulating mitochondrial DNA levels are inversely related to placenta oxygen partial pressure in the umbilical vein and directly related to and therefore a potential early biomarker for intra-uterine growth restriction (IUGR) as well as pre-eclampsia [7,9]. Interestingly, our data showed a statistically significant increase in mtDNA in placental blood (T3). Considering that placental dysfunction such as IUGR and pre-eclampsia as well Triple I correlates well with increased mtDNA levels, this biomarker may be more specific to detecting Triple I compared to classical biomarkers.

Study limitations include a small sample size, which did not allow us to account for regression analysis and find a threshold for circulating cell-free mitochondrial DNA as a predictive IAI biomarker. Another limitation is the selection of a control group consisting of pregnant women at later gestational ages who were undergoing routine blood checks. The lack of perfect gestational age matching between groups may affect the study’s interpretative power. Despite inconclusive results published concerning mtDNA levels in late pregnancy, the mtDNA levels in our intervention group were several times higher than those observed in both studies in early and late pregnancies and non-pregnant women by both research groups [7,8].

Additionally, the present study did not assess the origin of cell-free mtDNA experimentally; thus, it is currently uncertain whether circulating cell-free mitochondrial DNA is released in the maternal circulation during pregnancy by maternal or fetal tissues. 

## 5. Conclusions

In conclusion, circulating cell-free mtDNA is increased in women affected by IAI and may be a useful biomarker in the prediction and diagnosis of IAI. Future research is needed to determine reference values for the sensitivity of predicting IAI.

## Figures and Tables

**Table 1:** Basic patient characteristics of IAI and control groups.

	IAI *n* (%) or Mean ± SD	Control *n* (%) or Mean ± SD	*p*-Value
Patients	12 (100%)	21 (100%)	ns
Maternal age (years)	32.08 (5.47)	32.14 (5.30)	ns
Maternal weight (kg)	74.92 (14.85)	79.72 (11.81)	ns
Maternal BMI	27.44 (5.81)	29.77 (4.55)	ns
Nicotine	-	-	
yes		4 (19%)	
no	12 (100%)	17(81%)	
Neonatal birth weight (grams)	911.3 (231)	3243 (491)	<0.0001
GA at timepoint 1 (compl. weeks)	24.75 (0.97)	36.14 (0.48)	<0.0001
GA at delivery (compl. weeks)	26.25 (1.55)	38.05 (0.50)	<0.0001

Categorical data are presented as frequency and percentage. Continuous variables are expressed as means ± SD. BMI: pre-pregnancy body mass index, GA: gestational age in completed weeks.

## Data Availability

The datasets used and/or analyzed during the current study are available from the corresponding author on reasonable request.

## References

[1] Malik A.N., Czajka A. (2013). Is mitochondrial DNA content a potential biomarker of mitochondrial dysfunction?. Mitochondrion.

[2] Malik A.N., Shahni R., Rodriguez-de-Ledesma A., Laftah A., Cunningham P. (2011). Mitochondrial DNA as a non-invasive biomarker: Accurate quantification using real time quantitative PCR without co-amplification of pseudogenes and dilution bias. Biochem. Biophys. Res. Commun..

[3] Gray M.W. (2012). Mitochondrial evolution. Cold Spring Harb. Perspect. Biol..

[4] Taanman J.W. (1999). The mitochondrial genome: Structure, transcription, translation and replication. Biochim. Biophys. Acta.

[5] Krychtiuk K.A., Ruhittel S., Hohensinner P.J., Koller L., Kaun C., Lenz M., Bauer B., Wutzlhofer L., Draxler D.F., Maurer G. (2015). Mitochondrial DNA and Toll-Like Receptor-9 Are Associated with Mortality in Critically Ill Patients. Crit. Care Med..

[6] Zhang Q., Raoof M., Chen Y., Sumi Y., Sursal T., Junger W., Brohi K., Itagaki K., Hauser C.J. (2010). Circulating mitochondrial DAMPs cause inflammatory responses to injury. Nature.

[7] Colleoni F., Lattuada D., Garretto A., Massari M., Mandò C., Somigliana E., Cetin I. (2010). Maternal blood mitochondrial DNA content during normal and intrauterine growth restricted (IUGR) pregnancy. Am. J. Obstet. Gynecol..

[8] Cushen S.C., Sprouse M.L., Blessing A., Sun J., Jarvis S.S., Okada Y., Fu Q., Romero S.A., Phillips N.R., Goulopoulou S. (2020). Cell-free mitochondrial DNA increases in maternal circulation during healthy pregnancy: A prospective, longitudinal study. Am. J. Physiol. -Regul. Integr. Comp. Physiol..

[9] Marschalek J., Wohlrab P., Ott J., Wojta J., Speidl W., Klein K.U., Kiss H., Pateisky P., Zeisler H., Kuessel L. (2018). Maternal serum mitochondrial DNA (mtDNA) levels are elevated in preeclampsia—A matched case-control study. Pregnancy Hypertens.

[10] Bradshaw J.L., Cushen S.C., Phillips N.R., Goulopoulou S. (2022). Circulating Cell-Free Mitochondrial DNA in Pregnancy. Physiology.

[11] Higgins R.D., Saade G., Polin R.A., Grobman W.A., Buhimschi I.A., Watterberg K., Silver R.M., Raju T.N., Chorioamnionitis Workshop Participants (2016). Evaluation and Management of Women and Newborns with a Maternal Diagnosis of Chorioamnionitis: Summary of a Workshop. Obstet. Gynecol..

[12] Committee on Obstetric Practice (2017). Committee Opinion No. 712: Intrapartum Management of Intraamniotic Infection. Obstet. Gynecol..

[13] Yoon B.H., Romero R., Bin Moon J., Shim S.-S., Kim M., Kim G., Jun J.K. (2001). Clinical significance of intra-amniotic inflammation in patients with preterm labor and intact membranes. Am. J. Obstet. Gynecol..

[14] Kim C.J., Romero R., Chaemsaithong P., Chaiyasit N., Yoon B.H., Kim Y.M. (2015). Acute chorioamnionitis and funisitis: Definition, pathologic features, and clinical significance. Am. J. Obstet. Gynecol..

[15] Goldenberg R.L., Hauth J.C., Andrews W.W. (2000). Intrauterine infection and preterm delivery. N. Engl. J. Med..

[16] Lau J., Magee F., Qiu Z., Houbé J., Von Dadelszen P., Lee S.K. (2005). Chorioamnionitis with a fetal inflammatory response is associated with higher neonatal mortality, morbidity, and resource use than chorioamnionitis displaying a maternal inflammatory response only. Am. J. Obstet. Gynecol..

[17] Ramsey P.S., Lieman J.M., Brumfield C.G., Carlo W. (2005). Chorioamnionitis increases neonatal morbidity in pregnancies complicated by preterm premature rupture of membranes. Am. J. Obstet. Gynecol..

[18] Rouse D.J., Landon M., Leveno K.J., Leindecker S., Varner M.W., Caritis S.N., O’Sullivan M.J., Wapner R.J., Meis P.J., Miodovnik M. (2004). The maternal-fetal medicine units cesarean registry: Chorioamnionitis at term and its duration—Relationship to outcomes. Am. J. Obstet. Gynecol..

[19] Hauth J.C., Gilstrap L.C., Hankins G.D., Connor K.D. (1985). Term maternal and neonatal complications of acute chorioamnionitis. Obstet. Gynecol..

[20] Impey L., Greenwood C., MacQuillan K., Reynolds M., Sheil O. (2001). Fever in labour and neonatal encephalopathy: A prospective cohort study. BJOG Int. J. Obstet. Gynaecol..

[21] Helmo F.R., Alves E.A.R., Moreira R.A.d.A., Severino V.O., Rocha L.P., Monteiro M.L.G.d.R., dos Reis M.A., Etchebehere R.M., Machado J.R., Corrêa R.R.M. (2018). Intrauterine infection, immune system and premature birth. J. Matern. -Fetal Neonatal Med..

[22] Escames G., López L.C., García J.A., García-Corzo L., Ortiz F., Acuña-Castroviejo D. (2012). Mitochondrial DNA and inflammatory diseases. Hum. Genet..

[23] American College of Obstetricians and Gynecologists (2024). ACOG Clinical Practice Update: Update on Criteria for Suspected Diagnosis of Intraamniotic Infection. Obstet. Gynecol..

[24] Del Arroyo A., Sanchez J., Patel S., Phillips S., Reyes A., Cubillos C., Fernando R., David A., Reeve A., Sodha S. (2019). Role of leucocyte caspase-1 activity in epidural-related maternal fever: A single-centre, observational, mechanistic cohort study. Br. J. Anaesth..

